# Implications of controlled short-wavelength light exposure for sleep in older adults

**DOI:** 10.1186/1756-0500-4-334

**Published:** 2011-09-08

**Authors:** Mariana G Figueiro, Natalia Z Lesniak, Mark S Rea

**Affiliations:** 1 Lighting Research Center, Rensselaer Polytechnic Institute, 21 Union Street/3rd floor, Troy, NY, 12180, USA

## Abstract

**Background:**

Environmental and physiological conditions make older adults more likely to lose synchronization to their local time and experience sleep disturbances. A regular, 24-hour light/dark cycle promotes synchronization. It is now well established that the circadian system is maximally sensitive to short-wavelength (blue) light. The purpose of the present study was to measure dose effectiveness (amounts and durations) of short-wavelength (blue) light for stimulating the circadian systems of older adults. We investigated the impact of six corneal irradiances (0.7 to 72 μW/cm^2^) of 470-nm light on nocturnal melatonin production. Nine participants, each over 50 years of age completed a within-subjects study. Each week, participants were exposed to one of the six irradiances of 470-nm light for 90 minutes.

**Findings:**

A two-factor (6 corneal irradiances × 10 exposure durations), within-subjects analysis of variance (ANOVA) was conducted using the melatonin suppression levels. The ANOVA revealed a significant main effect of corneal irradiance (F_5, 30 _= 9.131, p < 0.0001), a significant main effect of exposure duration (F_9, 54 _= 5.731, p < 0.0001), and a significant interaction between these two variables (F_45,270 _= 1.927, p < 0.001). Post hoc t-tests revealed that corneal irradiances as low as 2 μW/cm^2 ^reliably suppressed melatonin after 90-minute exposure whereas 0.7 μW/cm^2 ^did not.

**Conclusions:**

Sleep disorders are common and a serious problem for millions of older adults. The present results showed that comfortable, precise and effective doses of light can be prescribed to older adults to reliably stimulate the circadian system that presumably would promote entrainment and, thus, regular sleep. Field studies on the impact of short-wavelength-light doses on sleep efficiency in older adults should be performed.

## Introduction

A large and growing body of research shows that a regular, 24-hour light/dark cycle synchronizes circadian rhythms in all species, including humans, to local time on earth. Light levels required to activate the circadian system are higher than those required for vision [1]. Since older adults are less active and tend to stay indoors more often where light levels tend to be low, they may not be exposed to lighting that sufficiently stimulates their circadian system [2]. Moreover, these same older adults commonly have less light reaching their retina due to natural age-related reductions in pupil size (senile miosis) and in crystalline lens transmission [3]. Together, these environmental and physiological conditions make older adults more likely to lose entrainment to their local time and, as a result, more likely to experience sleep disturbances [2,4,5].

Timed light treatments have been shown to improve circadian entrainment in older persons, including those with Alzheimer's disease (AD), a population that is perhaps most susceptible to circadian disruption through light restriction [6]. Clinical research has shown, for example, that exposing persons with AD to light levels higher than what they would normally experience in their homes or in institutions during the day and darkness at night can consolidate their rest/activity patterns [7,8]. Morning and evening bright light exposures (> 2,500 lux at the cornea) have been shown to improve nighttime sleep, increase daytime wakefulness, reduce evening agitation behavior, and consolidate rest/activity patterns of persons with AD [8-11]. Riemersma-van der Lek and colleagues demonstrated that all-day exposure to high levels of a white light (i.e., at least 1000 lux at the eye of a 4100 K light source) improved sleep efficiency and cognition in patients with AD as well as reduced symptoms of depression [12]. Light applied in the evening has also been shown to be effective in phase delaying the circadian system of older people without dementia, allowing them to fall asleep later and wake up later [13].

Although bright light interventions appear to be effective for promoting circadian entrainment and improving sleep efficiency, they can be expensive, time consuming for caregivers, and uncomfortable for older adults, who show symptoms of photophobia. In fact, there is evidence that light treatment may not always result in better sleep in older adults. Light treatment alone (1 hour of 2,500 lux at the angle of gaze) did not improve nighttime sleep, daytime wake and rest activity in AD patients [14,15]. These negative results may reflect problems with compliance, so there is a need to develop more practical and, thus, more effective methods for light delivery.

Treatment efficacy (i.e., effectiveness per electrical watt) is also important to consider for practical applications. One simple method for improving treatment efficacy is placing the light source closer to the eyes. The irradiance at the cornea falls off with the square of the distance from the source, so small reductions in this distance have disproportionately large increases in light exposure to the retina. Tuning the light spectrum for maximum effect also increases treatment efficacy. Research has demonstrated that the spectral sensitivity of the circadian system differs from the spectral sensitivity of the achromatic visual channel, which is used for such visual activities as reading. Short-wavelength (blue) light between approximately 440 nm and 470 nm is maximally effective at stimulating the human circadian system, as measured through acute melatonin suppression and phase shifting of the melatonin rhythm [16-18].

One way to provide older adults with inexpensive, low-powered light sources that provide sufficient levels of circadian-effective light would be to mount short-wavelength light-emitting diodes (LEDs) on spectacle frames. With a thoughtful design, the intensity distribution of the circadian-effective light sources should not interfere with the performance of visual tasks. Some engineering sophistication is also needed to precisely control the timing and duration of the light dose. Temporally controlled 470-nm (blue) LEDs mounted on spectacle frames would be inexpensive, require little, if any, maintenance, and would be comfortable and safe for users as long as the dose was properly prescribed. A prototype of the device was developed and its effectiveness at two irradiances was measured previously by Figueiro et al [19].

The purpose of the present study was to extend the findings from Figueiro et al [19] to more precisely measure dose effectiveness (amounts and durations) of short-wavelength (blue) light for stimulating the circadian systems of older adults. A self-powered, blue-light goggle system had been developed for the previous study and was used again here to deliver the light doses. Nocturnal melatonin suppression was used as the measure of the threshold for light activation and of dose efficacy. In addition, these data would be expected to be used to refine the mathematical model of human circadian phototransduction [17] to predict circadian effectiveness for other light sources. Once the dose effectiveness for short-wavelength stimulus is known, the dose effectiveness for other narrowband sources can be estimated from the modeled spectral sensitivity function.

## Methods

The prototype blue-light goggles were developed under a grant from the National Institutes on Aging to deliver circadian-effective light doses to adults 50 years of age and older. Previously, nocturnal melatonin suppression was measured at just two light doses from these blue-light goggles [19]. The present study was designed to extend those findings by measuring the effectiveness of additional doses from these goggles for suppressing nocturnal melatonin in older adults. The Institute Review Board (IRB) of Rensselaer Polytechnic Institute approved the present experimental protocol and all subjects were asked to sign a written informed consent form.

### Subjects

Of the eleven subjects who completed the previous study [19], nine were still available for further study; therefore only data for the nine subjects who completed all experimental conditions are included here. These subjects had been previously recruited through e-mail notices, posters, and word-of-mouth. Eligibility for the study required subjects to be over the age of 50 years and to be free of any major health problems, such as cardiovascular disease, diabetes, or high blood pressure. Potential subjects were excluded if they were taking over-the-counter melatonin or prescription medication such as blood pressure medicine, antidepressants, sleep medicine, hormone replacement therapy or beta-blockers. Potential subjects who stated they had any eye disease (such as cataract, glaucoma and color blindness) were not accepted into the study. Seven of nine subjects completed the Munich ChronoType questionnaire (MCTQ) [20]. Because the experiment would be conducted in the early part of the night, extreme late chronotypes were excluded to assure melatonin levels would be high at the time of the experiment; of the seven subjects who completed the MCTQ, all were moderate to early chronotypes with a mean ± standard deviation of 1.3 ± 1.0. All subjects contributing to the present results were between 51 and 62 years of age (mean ± standard deviation, 56.2 ± 3.9 years).

### Protocol

Subjects who completed the 2-night protocol reported by Figueiro et al [19] took part in the additional five-night protocol; the sessions took place on Friday nights (between September 2009 and April 2010) and were scheduled at least one week apart. On the weeks of the experimental sessions, subjects were instructed to go to bed between 21:00 and 23:00. On the day of the experiment, subjects were asked to refrain from caffeine and alcohol 12 hours prior to the start of the experiment and to arrive at the lab at 22:00. Upon arrival, a registered nurse inserted an indwelling catheter into an arm of each subject. At 23:00, room lights were turned off, except for two red LED traffic signal lights (λ_max _= 640 nm) placed on the floor, outside the subjects' field of view. These traffic signals provided indirect illumination in the space, less than 1 lux at the cornea, that was sufficient for people to safely navigate in the space without stimulating their circadian systems.

After the room lights were turned off, the subjects were seated and watched a movie that was projected on a screen about 20 feet (6 meters) away. The measured (polychromatic) illuminance at the subjects' cornea reflected from the projection screen alone ranged from 0.14 to 0.2 lux; this level is too low to stimulate the circadian system [21]. The movie kept subjects awake, minimizing the likelihood of eye closures. Moreover, experimenters continuously monitored the subjects to ensure that they had their eyes open. Subjects were not allowed to drink or eat after 23:30.

Table 1 shows the times that blood and saliva samples were taken from the first subject in a session. To allow the nurse to collect the blood samples from every subject in a session, data collection times for the other subjects were each staggered by about 2 minutes. The first two blood and saliva samples were collected at the same time (23:50 and 00:00). Immediately after the second sample was collected from the subjects, they were asked to put on the blue-light goggles (described below), which remained on for the remainder of the experimental session.

**Table 1:** Data collection times for the first subject in a session to provide blood and saliva samples

	Dark	Dark	Goggles on	Goggles on	Goggles on	Goggles on	Goggles on
Blood	23:50	00:00	00:10	00:20	00:30	01:00	01:30
Saliva	23:50	00:00	00:05	00:15	00:25	00:45	01:15

One saliva and three blood samples were collected from each subject at each of seven appointed times in a 90-minute session. Saliva samples were obtained using the Salivette system (ALPCO Diagnostics, Salem, NH, USA). Three, 3-ml blood samples were drawn by the nurse at each of the prescribed times; the first sample was always discarded and the second and third samples were spun in a centrifuge for 15 minutes. Frozen saliva and plasma samples were subsequently sent to an independent laboratory (Pharmasan Labs, Osceola, WI) for melatonin radioimmunoassay. The sensitivity of the saliva assay was 0.7 pg/ml and the intra- and inter-assay coefficients of variability (CVs) were 12.1% and 13.2%, respectively. The sensitivity of the plasma assay was 3.5 pg/ml and the intra- and inter-assay CVs were 8.1% and 14.8%, respectively.

All subjects saw all lighting conditions described in Table 2 in a counterbalanced order within a set of sessions. Thus, different light exposures were provided to subjects on any given night. When experiencing the dark (control) condition, subjects wore non-energized blue-light goggles. Melatonin concentrations in saliva and in plasma were assayed at different times as three different batches, also shown in Table 2.

**Table 2:** Lighting conditions presented to subjects

Batch	Irradiance (μW/cm^2^)	Illuminance (lux)	Peak wavelength (nm)	FWHM (nm)	Pupil diameter (mm)
3	dark	NA	NA	NA	5.5 (5.4 ± 0.51)

3	0.7 (0.7 ± 0.04)	0.61(0.6 ± 0.16)	473 (473 ± 0.4)	18 (18 ± 0.83)	5.3 (5.2 ± 0.52)

3	2.0 (2 ± 0.08)	1.2 (1.5 ± 0.37)	462 (467 ± 5.5)	18 (18 ± 0.29)	4.9 (4.8 ± 0.53)

2	6.1 (6 ± 0.2)	5.8 (6.8 ± 3.1)	474 (474 ± 0.3)	20 (20 ± 0.3)	4.2 (4.4 ± 0.61)

1	11 (11 ± 2.6)	11 (11 ± 1.6)	474 (474 ± 1.8)	20 (20 ± 0.5)	3.8 (4.1 ± 0.91)

2	20 (20 ± 0.4)	12 (14 ± 4.1)	462 (466 ± 5.9)	19 (19 ± 1.1)	3.8 (4.1 ± 0.87)

1	72 (74 ± 7.3)	66 (60 ± 17)	473 (469 ± 6.3)	20 (20 ± 1.5)	3.4 (3.4 ± 0.33)

Medians and, in parentheses, means ± standard deviations of corneal irradiance, corneal illuminance, peak wavelength and full width at half maximum (FWHM) wavelength delivered by the blue-light goggles used in the experiment. Peak wavelengths vary slightly because different goggles were used to deliver the different light levels. Medians, means ± standard deviations of measured subject pupil diameter while wearing the blue-light goggles at the prescribed levels are also provided. Pupil areas and corneal irradiances or corneal illuminance levels were used to estimate retinal light doses. Melatonin concentrations in saliva and in plasma were assayed at different times as three different batches.

At completion of pupil measurements (described below), room lighting was turned on and the registered nurse removed the indwelling catheter from the subjects' arms and subjects were offered a ride home.

### Light Levels

Topbulb.com produced the blue-light goggles used to deliver the prescribed doses of light from LUXEON Rebel (LXML - PB01) LEDs. Subjects wore the goggles while they received one of seven irradiance levels at the cornea, including the dark control condition without energizing the LEDs. Table 2 shows the six average irradiance (μW/cm^2^) and illuminance (lux) levels, as well as the peak and full width at half maximum (FWHM) wavelengths generated by the energized goggles.

Four LEDs, two per goggle lens, with peak wavelengths ranging from 466 to 474 nm were mounted to the top inside rim of the goggles. The LEDS were located behind a translucent plastic tube to diffuse the light and to reduce point-source glare [22]. Although the amount of light emitted by the LEDs was below levels that might be of concern for blue light hazard [23], diffusing the light sources further ensured safety. Heat from the LEDs was dissipated via a heat sink strip. The LEDs were connected by wire to a small control case (0.5 × 1.5 × 2.5 in. [1.3 × 3.8 × 6.4 cm]), which held a 3.0 VDC two-battery power source, and the control circuitry, including a Texas Instruments voltage regulator (TPS77018DBVR 1.8 V). The small control case also housed the LED driver (Maxim Evaluation Kit -1848 EVKIT), which was used to adjust the flux emitted by the LEDs to the prescribed light level in a session.

Prior to every session, new batteries were inserted into the small control case. While the LED driver was adjusted, light from the left and from the right goggle lens LEDs were alternately measured with a Model 2300i Action Research double monochrometer a Spectra Sense Version 4.3.0 spectral radiometer using a 5 mm diameter optical fiber ending in a lambertian diffuser as the sensor. During measurements, the sensor was placed where the subject's corneas would be while wearing the goggles. The average irradiance from the left and right lenses was used to meet the prescribed light level.

After each session, while a subject continued to wear the blue-light goggles at the session-prescribed corneal irradiance, ten digital images of one eye of each subject were acquired with a Sony video camera. To improve camera image quality, an infrared (IR) source irradiated the eye. Pupil size is unaffected by this supplemental source because the retina is insensitive to IR radiation. Subjects held a small ruler just below their eye during the image capture as a reference, linear scale to estimate pupil diameter. The captured image was printed and, using precise calipers, the experimenter measured both the size of the known ruler unit (e.g., 1 cm) and the size of the unknown pupil diameter in the image. The ratio of the measured ruler unit from the digital image to the known ruler unit was then used to scale the measured pupil diameter from the digital image to estimate the actual pupil diameter. Three images were arbitrarily selected and used to estimate the pupil diameter for a given subject. Thus, the reported median pupil diameters in Table 2 are each based upon the median of 27 pupil diameter estimates (9 subjects × 3 samples). From the median pupil diameters, pupil areas were calculated. Since there was no evidence of anisocoria with any subject, the size of both the left and right pupils should always be the same. Binocular retinal light exposures were defined as the product of the median corneal irradiance and the median pupil area in units of deka-nanowatts (μW/cm^2 ^× mm^2^) or micro-lumens (lm/m^2 ^× mm^2^) where corneal illuminance is considered.

### Data Analyses

**Figure 1:**
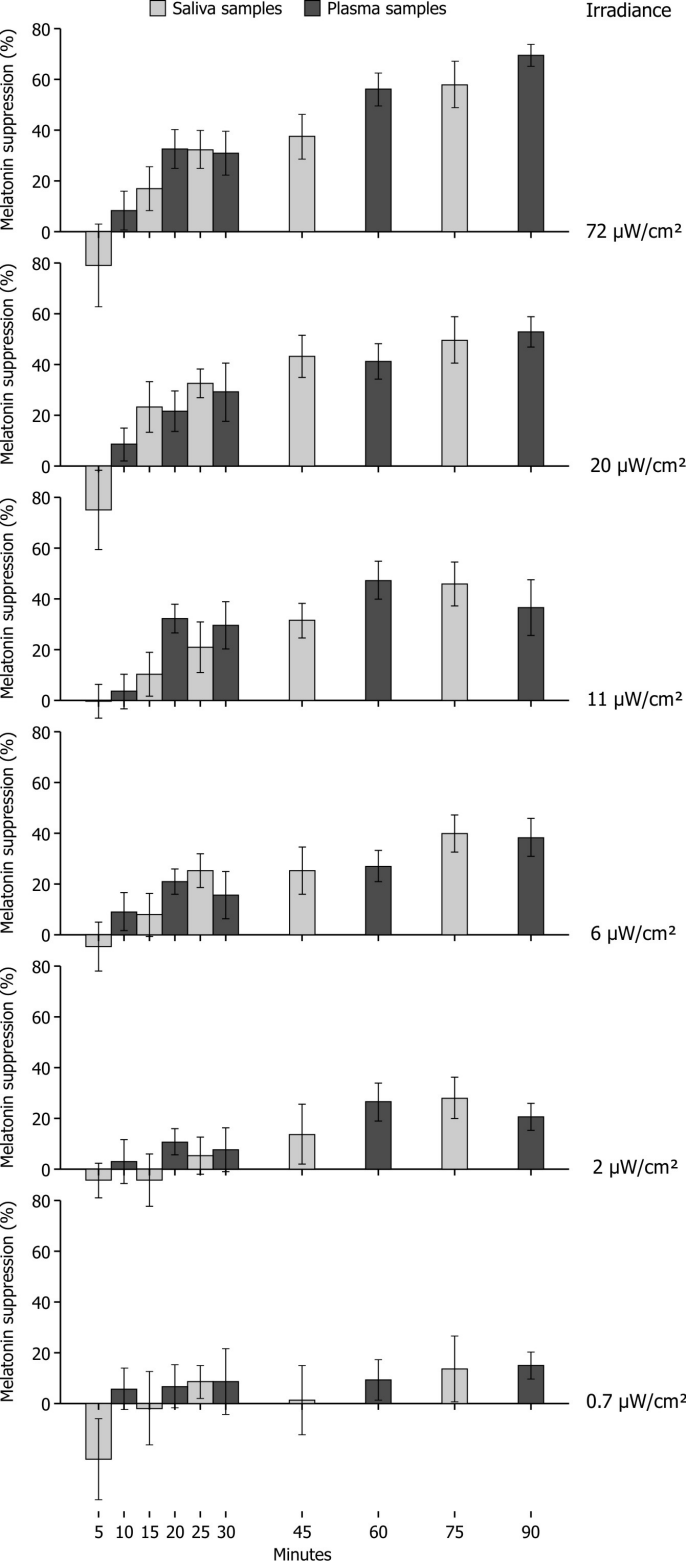
**Melatonin suppression for saliva and plasma samples**.

To more precisely determine the differential effects of light exposure level and duration on melatonin concentrations a series of normalizations were undertaken to minimize systematic differences among the nine subjects, between the two biomarker types (plasma and saliva), and among assay batches (Table 2). This approach was the same as that employed in the previous study by Figueiro and colleagues [19], except for the additional normalization for assay batches. Although the assay methodology was not changed, the absolute melatonin concentrations differed systematically among the three batches sent for melatonin assays as revealed by examination of the saliva and plasma concentrations at 23:50 and 00:00, the two times, in the dark, common to all experimental sessions. First, the raw melatonin concentrations in a batch, *either *plasma *or *saliva, were averaged, and the ratio of the batch average concentrations to the grand average concentrations were used to normalize the different batches for plasma and saliva concentrations. The same procedure was then followed to normalize differences among subjects. Thus, any systematic differences among batches, among subjects, and between plasma and saliva melatonin concentrations were minimized. The subject's normalized melatonin concentrations, both plasma and saliva, for every exposure duration at a given retinal irradiance were then fitted with a polynomial equation using a least squares criterion. Each of those resulting normalized melatonin concentration curves, one for every subject at every retinal irradiance, including dark, were then adjusted to a common melatonin concentration value at 00:00; the adjustment was based upon the reciprocal of the polynomial values at 00:00 for combined normalized plasma and saliva data. Melatonin suppression for each subject (ms_n_%) was then calculated from the fitted melatonin concentration values at each retinal irradiance and for all exposure durations; the mean melatonin suppression (ms%) levels shown in Figure 1 were based upon mean suppression levels from every subject at each retinal irradiance and every exposure duration. Suppression levels for a given subject were determined by the ratio of the melatonin concentrations after exposure to a given retinal irradiance for a given duration after 00:00 to the melatonin concentrations during the dark night at the same clock time.

## Results

### Melatonin Suppression

Figure 1 shows mean nocturnal melatonin suppression levels following exposure to each retinal irradiance level at every exposure duration for both the plasma and the saliva samples. A two-factor (6 corneal irradiances × 10 exposure durations), within-subjects analysis of variance (ANOVA) was conducted using the melatonin suppression levels (SPSS version 13). The ANOVA revealed a significant main effect of corneal irradiance (F_5, 30 _= 9.131, p < 0.0001), a significant main effect of exposure duration (F_9, 54 _= 5.731, p < 0.0001), and a significant interaction between these two variables (F_45,270 _= 1.927, p < 0.001). Table 3 shows the results of 60 post hoc, two-tailed one sample t-tests to determine if the suppression levels were significantly different than zero. Entries in the table are the independent probabilities of a Type 1 error. The Bonferonni-adjusted criterion probability of a Type 1 error is 0.0008; probabilities less than this criterion are marked with asterisks in Table 3. The tendency for significant post hoc comparisons to lie in the bottom right corner of Table 3 illustrates the significant interaction between exposure duration and corneal irradiance.

**Table 3:** Probabilities of a Type 1 error using one sample Student's t-test

		Corneal irradiance (μW/cm^2^)					
		
Sample type	Exposure duration (min)	0.7	2	6	11	20	72
saliva	5	0.210	0.523	0.634	0.955	0.303	0.442
plasma	10	0.504	0.744	0.266	0.630	0.226	0.306
saliva	15	0.896	0.688	0.386	0.273	0.0520	0.0950
plasma	20	0.455	0.0768	0.00292	0.00048*	0.0259	0.0030
saliva	25	0.231	0.504	0.00666	0.0725	0.0008*	0.0033
plasma	30	0.533	0.414	0.133	0.0134	0.0353	0.0075
saliva	45	0.935	0.284	0.0308	0.00262	0.0021	0.0039
plasma	60	0.280	0.0078	0.00232	0.00021*	0.0004*	0.0000*
saliva	75	0.329	0.0110	0.0009	0.0011	0.0009	0.0004*
plasma	90	0.0246	0.0042	0.0009	0.0109	0.0000*	0.0000*

*- statistically different than zero suppression using the Bonferroni correction for multiple t-tests.

**Figure 2:**
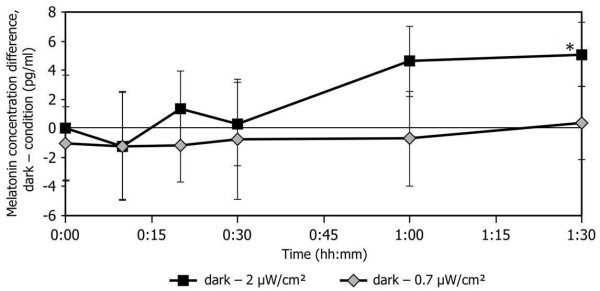
**Estimation of threshold dose at different exposure durations using the difference between melatonin concentrations in the dark and at 0.7 μW/cm^2 ^as well as the difference between melatonin concentrations in the dark and at 2 μW/cm^2^**. The asterisk indicates a statistically reliable difference at p = 0.05.

In order to estimate threshold values for melatonin suppression by the 470-nm light, we compared the melatonin concentrations in the dark to those after exposure to 0.7 μW/cm^2 ^and 2 μW/cm^2^. Figure 2 shows a plot of the mean differences between the measured plasma melatonin concentrations in the dark and those at both 0.7 μW/cm^2 ^and 2 μW/cm^2 ^at the same exposure durations. These data were used because melatonin assays for these three corneal irradiance conditions were performed as a single batch. Post hoc 2-tailed t-tests were performed to verify if the difference values were reliably different than zero. The melatonin concentration differences between dark and 2 μW/cm^2 ^were only significantly different than zero at the 90-minute exposure duration (p = 0.05), although the differences at the 60-minute exposure duration were almost statistically reliable (p = 0.09). The melatonin concentration differences between dark and 0.7 μW/cm^2 ^were not significantly different than zero at any exposure duration, indicating that 0.7 μW/cm^2 ^of 470 nm is very close to or at threshold for nocturnal melatonin suppression for exposure durations up to and including 90 minutes (Figure 2).

## Discussion

The present results investigated the impact of 90-minute exposures from six corneal irradiances (0.7 to 72 μW/cm^2^) of 470-nm (blue) light on acute nocturnal melatonin suppression. In general, as irradiances and duration of exposure increased, melatonin suppression increased. The present data also showed that statistically significant melatonin suppression was observed after 90-minute exposure to 2 μW/cm^2^, suggesting that the threshold for acute suppression by a 470-nm light is between 0.7 μW/cm^2 ^and 2 μW/cm^2 ^for this population.

Sleep disorders are common and a serious problem for millions of older adults [24-26]. Family members, friends, and health care workers who provide care to these individuals are also severely impacted by sleep disorders. Pharmaceuticals are commonly used to treat sleep problems with mixed success and at a fairly high cost. Light therapy to correct circadian disruption may be a practical, inexpensive, and an effective treatment alternative for older adults with sleep disorders. The present data, together with a model of human circadian phototransduction [17], enables researchers to make refined quantitative predictions of light doses and clinicians to more precisely prescribe light doses to treat circadian sleep disorders in older adults.

Administering light through a personal device does not require the person to be seated in front of a light box for a long period of time. Individuals can wear the device while performing routine tasks in the home. As shown here, although no formal collection protocol was used, informal discussions with subjects at the end of the experiment revealed that they could comfortably watch a movie in a darkened room while receiving their light dose. As a result of tuning the temporal-spatial-spectral-absolute characteristics of light to the human circadian system, it is possible to prescribe comfortable and effective doses of light to reliably stimulate the circadian system. This tuning reduces glare and discomfort associated with bright white light and should thereby increase light treatment compliance. Simple control technologies, yet to be developed, would ensure that the light delivery is provided at the right time and for the shortest duration to promote circadian entrainment. The personal light treatment device is inherently inexpensive to fabricate, calibrate, and obviates replacing or retrofitting existing light fixtures to deliver an effective dose of light. This is especially important for many assisted living facilities or nursing homes with low budgets.

In summary, the present study was able to quantify the efficacy of a personal light treatment device for stimulating the circadian systems of older adults without risk of retinal damage [23]. In fact, the radiances utilized in the study were well below levels that pose a blue-light hazard risk [23]. Although the present data were collected in the middle of the night, this device should be used to deliver light treatment during the daytime or during the early evening hours. Additional studies are recommended to investigate the ability of short-wavelength (blue) light applied during the morning or evening hours to phase shift or to entrain the circadian systems of older adults suffering from circadian sleep disorders. Additional studies are also recommended to investigate the impact of blue-light treatment for subjective daytime sleepiness and nighttime sleep quality as well as for wake and sleep electroencephalogram. Notwithstanding these important limitations of the present study, the primary envisioned benefit of a personal light treatment device is that clinicians can begin to prescribe a precise dose of short-wavelength (blue) light for older adults to reliably stimulate their circadian systems [27]. Once a dose is determined, its brightness would be the lowest possible because the spectrum is tuned to maximum circadian effectiveness. This also has the benefit of limiting wasted heat from the LED, both improving energy efficiency and minimizing tactile discomfort. A low-brightness, battery-operated device that is safe, comfortable, and portable can provide older adults with an effective light dose while they perform their daily activities, eliminating the need to sit in front of a light box.

Two further limitations of the present study are that user acceptance was not formally evaluated and the effectiveness of blue light exposures was not tested in older adults who exhibit health problems. Although the efficacy of blue-light treatment for improving sleep efficiency in older adults, including those with AD, has been demonstrated in earlier studies [27,28], there are no empirical data to directly support acceptance, compliance, or efficacy for treatment of sleep disorders in larger clinical trials using older adults who are not as healthy as those who participated in the present study. Further, the device used in the present study is not yet a commercial product so customer feedback in other areas, like cost, was not obtained. Still, the device seems quite promising for formal tests of user acceptance.

## Competing interests

The authors declare that they have no competing interests.

## Authors' contributions

MGF participated in the design of the experiment, data collection, data analyses and drafted the manuscript. MSR participated in the design of the experiment, data analyses and manuscript writing. NZL participated in data collection and analyses and contributed to manuscript writing and review. All authors read and approved the final manuscript.
